# Temporal trends in the prevalence of Alzheimer's disease, 1980–2024: a systematic review and meta-analysis

**DOI:** 10.3389/fpubh.2026.1884058

**Published:** 2026-08-19

**Authors:** Mengqi Hu, Zhiyuan Sun, Dewei Mao, Xuewen Tian, Qinghui Shang

**Affiliations:** 1 Shandong Sports Science Research Institute, Shandong Sport University, Jinan, Shandong, China; 2 Division of Physical Education, The Chinese University of Hong Kong, Shenzhen, Guangdong, China

**Keywords:** Alzheimer's disease, epidemiology, prevalence, systematic review, temporal trends

## Abstract

**Background:**

Alzheimer's disease (AD) is the primary cause of dementia and represents a significant public health concern in aging populations. Existing evidence indicates geographic and sex-related differences in AD prevalence; however, comparability across time periods and diagnostic criteria is limited, and data from lower-resource regions remain scarce. This study synthesized evidence on reported AD prevalence from 1980 to 2024 and examined temporal, geographic, demographic, and methodological variation.

**Methods:**

PubMed, Web of Science, and Embase were systematically searched from inception to December 31, 2024, for observational studies reporting AD prevalence in general or community-based populations. Eligible studies reported prevalence estimates with 95% confidence intervals or provided sufficient data for calculation. Studies with sample sizes below 100 and non-original reports were excluded. Two reviewers independently screened studies and extracted data; disagreements were resolved by a third reviewer. Pooled reported prevalence was estimated using a random-effects model. The review was prospectively registered in PROSPERO (CRD420251111597).

**Results:**

Fifty-two studies from 19 countries met the inclusion criteria. The pooled reported prevalence of AD was 4.43 per 100 population (95% CI 3.47–5.50). Prevalence was higher among females (4.65 per 100, 95% CI 3.37–6.13) than males (2.30 per 100, 95% CI 1.71–2.97). By survey period, reported prevalence was 3.52 per 100 for 1980–1989, 4.21 for 1990–1999, 4.12 for 2000–2009, and 6.78 for 2010–2024. Adjusted meta-regression did not identify a significant association between survey year and reported prevalence. Estimates also varied by WHO region, Human Development Index (HDI) level, sample size, and study design.

**Conclusions:**

Reported AD prevalence was substantial and varied by demographic, geographic, and methodological factors. Although prevalence was highest in the most recent survey period, meta-regression did not indicate a consistent global increase over time. Given the extremely high between-study heterogeneity, these findings should be interpreted with caution. More standardized, age-comparable, and geographically representative studies are needed, particularly in underrepresented and lower-resource settings.

**Systematic review registration:**

https://www.crd.york.ac.uk/PROSPERO/view/CRD420251111597, identifier CRD420251111597.

## 1 Introduction

Dementia represents a significant public health concern as the global population ages. The World Health Organization (WHO) estimates that approximately 57 million individuals are currently affected by dementia, with nearly 10 million new cases diagnosed annually. Projections indicate that this number will increase to about 78 million by 2030 and may reach approximately 139 million by 2050 (1). As the proportion of older adults rises, the associated challenges are expected to intensify, placing substantial strain on healthcare systems, social support structures, and family caregiving capacities.

Alzheimer's disease (AD) is the primary cause of dementia, accounting for approximately 60% to 70% of all cases worldwide (2). This neurodegenerative disorder is characterized by a progressive decline in cognitive functions and neuronal damage. In the early stages, individuals with AD typically experience impairments in memory, language, and cognitive processing. As the disease progresses, neuronal damage spreads to additional brain regions, leading to alterations in emotions, behavior, and personality. This progression can severely impede essential daily activities, including walking and swallowing, often necessitating continuous care (3). Consequently, AD profoundly diminishes the quality of life for affected individuals and imposes substantial economic and social burdens on families and communities (4).

In light of the growing global challenge posed by dementia, it has become essential to pinpoint modifiable risk factors and create effective strategies for prevention. Research has shown that the risk of developing dementia is linked to various modifiable factors throughout an individual's life. For instance, early educational experiences can enhance cognitive reserve, while factors related to vascular health, metabolism, and lifestyle choices in middle and later life can increase risk. Additionally, there are significant disparities in diagnostic capabilities, healthcare access, and prevention efforts across different countries and regions (5). The 2024 update from the Lancet Commission highlighted that most existing research and intervention initiatives are heavily focused on high-income nations, leaving low- and middle-income countries and at-risk populations with limited access to evidence and resources. These disparities can hinder the effective identification, diagnosis, and treatment of dementia cases (6).

Beyond healthcare system disparities, sex-based variations in dementia and Alzheimer's disease (AD) epidemiology are critical for understanding disease impact. A comprehensive review and meta-analysis demonstrated that women generally exhibit a higher prevalence of AD than men across age groups. However, considerable heterogeneity among studies suggests that geographic variation, age distribution, and methodological differences may influence assessments of sex disparities (7).

Despite advances in research on dementia risk factors and prevention, substantial uncertainty remains regarding long-term trends in dementia prevalence. Prince et al., through a systematic review of studies from 1980 to the mid-2010s, reported that dementia incidence may have declined in high-income countries, while patient survival increased, resulting in inconsistent prevalence trends across studies (8). This indicates that overall epidemiological trends may not accurately capture the evolving characteristics of dementia subtypes. For Alzheimer's disease (AD) specifically, available studies are limited in temporal scope and population coverage. Additionally, variations in diagnostic criteria, diagnostic capacity, survey methodologies, and data collection across periods and regions further complicate prevalence estimation and cross-study comparisons (9).

This systematic review and meta-analysis synthesized evidence on the reported prevalence of Alzheimer's disease from 1980 to 2024. We aimed to estimate the pooled reported prevalence of AD across included studies, assess variation in reported prevalence by survey period, and examine differences by WHO region, Human Development Index (HDI), sex, sample size, data source, study design, and diagnostic criteria. By examining these sources of variation, this review sought to provide a comprehensive, methodologically rigorous summary of the available epidemiological evidence on AD prevalence.

## 2 Methods

### 2.1 Literature search strategy

This systematic review and meta-analysis was conducted in accordance with the PRISMA 2020 statement (10) (Supplementary Table S1). Comprehensive searches were performed across the PubMed, Embase, and Web of Science databases, covering the period from database inception to December 31, 2024. The search strategy incorporated both controlled vocabulary and free-text terms related to Alzheimer's disease, prevalence, epidemiology, and observational study designs. Detailed, database-specific search strategies are provided in Supplementary Text S1. Only English-language full-text publications were included, as eligibility assessment, diagnostic information extraction, and prevalence data verification could not be reliably conducted for studies published in other languages. Gray literature, conference abstracts, posters, letters, comments, and other non-full-text reports were excluded due to insufficient methodological detail or lack of extractable prevalence data. These restrictions are acknowledged as study limitations.

The search strategy was developed and implemented independently by Mengqi Hu and Zhiyuan Sun. Following duplicate removal, these reviewers independently screened records by title and abstract, then assessed full-text eligibility according to predefined inclusion and exclusion criteria. Discrepancies were resolved through discussion, with unresolved disagreements adjudicated by Dewei Mao.

### 2.2 Inclusion and exclusion criteria

Inclusion criteria were as follows: (1) original observational research studies; (2) populations derived from general or community-based groups or databases; and (3) availability of numerical data on the prevalence of Alzheimer's disease (AD), including reported 95% confidence intervals or sufficient information to calculate them. Exclusion criteria comprised: (1) studies with fewer than 100 participants; and (2) non-original research formats, such as review articles, case reports, study protocols, conference abstracts, letters to the editor, commentaries, short communications, posters, and similar reports. When multiple studies originated from the same cohort or population, the study providing the most detailed and current prevalence data for AD was selected. All identified literature was managed using Note Express software (version 3.8.0) to remove duplicate entries. The review was prospectively registered in PROSPERO (CRD420251111597). Minor protocol deviations occurred during the review process, including restricting the review to published studies, clarifying that eligible populations were adults aged 18 years or older, and omitting snowball sampling due to sufficient coverage from database searches. These deviations were documented and will be updated in the PROSPERO record.

### 2.3 Data extraction

A standardized template was used for data extraction, capturing the following domains: (1) publication characteristics, including first author and publication year; (2) study-level characteristics, such as country, World Health Organization (WHO) region, Human Development Index (HDI) (11), survey period, data source, study design, and diagnostic criteria; (3) participant characteristics, specifically sex and age range; and (4) prevalence data, including the number of Alzheimer's disease (AD) cases, total sample size, and prevalence estimates. HDI categories were assigned according to the United Nations Development Program classification, with definitions and categorizations provided in Supplementary Text S2. If prevalence was not explicitly reported, it was calculated from the available data. Studies utilizing different diagnostic criteria for Alzheimer's disease were classified into four mutually exclusive categories: Criterion 1, clinical diagnostic criteria; Criterion 2, International Classification of Diseases codes; Criterion 3, diagnosis based on clinical symptoms; and not-stated criteria. For studies reporting multiple diagnostic approaches, each was assigned to a single category to prevent double-counting. If clinical diagnostic criteria were included, the study was classified as Criterion 1, as this category represented the most clinically specific diagnostic basis. Detailed definitions of the diagnostic-criteria categories are provided in Supplementary Text S3.

### 2.4 Risk of bias assessment

The risk of bias in the included studies was evaluated using the Critical Appraisal Checklist for Prevalence Studies developed by the Joanna Briggs Institute. Study-level assessments are presented in Supplementary Table S2. This checklist comprises nine criteria, each rated as “yes,” “no,” “unclear,” or “not applicable.” For quantification, each “yes” response was assigned 1 point, while “no,” “unclear,” and “not applicable” responses received 0 points. The proportion of “yes” responses relative to the total number of items was calculated for each study. Studies were categorized based on these proportions: those with ≥80% “yes” responses were considered to have a low risk of bias (high-quality), those with 50%−80% were classified as moderate risk, and those with < 50% were deemed high risk of bias (low-quality). Risk of bias assessments were conducted independently by Mengqi Hu and Zhiyuan Sun. Discrepancies were resolved through discussion, with Dewei Mao serving as adjudicator when necessary. The overall confidence in the evidence was evaluated using the GRADE (Grading of Recommendations, Assessment, Development, and Evaluation) methodology (12). Detailed certainty assessments are provided in Supplementary Table S4.

### 2.5 Statistical analysis

Given the broad range of prevalence estimates and the presence of boundary values in the included studies, a random-effects model with the Freeman-Tukey double-arcsine transformation was employed to estimate the pooled reported prevalence of Alzheimer's disease (AD) and its 95% confidence interval. This method stabilizes variances and is appropriate for aggregating rates near boundary values. Application of the Freeman-Tukey double-arcsine transformation may result in asymmetric confidence intervals when back-transformed to the original scale.

For sensitivity analysis of the primary pooled reported prevalence estimate, the pooled prevalence was recalculated using a generalized linear mixed model with a binomial likelihood and logit link. The number of AD cases and the total sample size from each study served as model inputs, and the pooled estimate was back-transformed and reported as prevalence per 100 population.

Heterogeneity among studies was assessed using the Q and I^2^ statistics; values greater than 50% indicate substantial heterogeneity. Subgroup analyses explored potential sources of heterogeneity across categorical variables, including sex, survey period, WHO region, Human Development Index (HDI), sample size, study design, diagnostic criteria, and data source. To ensure comparability across subgroups, survey periods were categorized as 1980–1989, 1990–1999, 2000–2009, and 2010–2024. Subgroup differences were evaluated using chi-square tests for between-subgroup heterogeneity within the random-effects meta-analysis framework. These tests were conducted on the Freeman-Tukey double-arcsine transformed scale, while pooled prevalence estimates and 95% confidence intervals were back-transformed and reported as prevalence per 100 population. For main subgroup analyses, *p*-values indicate differences in pooled prevalence estimates across moderator categories. For nested subgroup figures, *p*-values reflect subgroup differences within each displayed stratum. In sex-specific subgroup figures, *p*-values represent male-female subgroup differences within each survey period, WHO region, or HDI category. No formal interaction tests were conducted; thus, these nested subgroup analyses were interpreted as exploratory.

To investigate the association between survey year and reported AD prevalence, random-effects meta-regression analyses were conducted. Survey year was included as a continuous moderator, defined as the midpoint of the reported survey period. For studies that span multiple survey years, the midpoint was calculated as the average of the start and end years. Studies lacking sufficient survey-year information were excluded from the meta-regression analyses. Univariable meta-regression was initially performed with survey year as the sole moderator, followed by a multivariable meta-regression model adjusted for WHO region, diagnostic criteria, and log-transformed sample size. Meta-regression analyses were conducted using the metafor package in R with restricted maximum likelihood estimation.

Sensitivity analyses were conducted to evaluate the reliability of the results by assessing the impact of excluding individual studies or removing low-quality studies on the overall effect size. Publication bias was assessed using funnel plots and Egger's test for funnel plot asymmetry (see Supplementary Figure S1).

All analyses were conducted using R software (version 4.4.3). The primary meta-analysis was performed with the meta package, while the GLMM sensitivity analysis and meta-regression analyses utilized the metafor package. A two-sided *p*-value less than 0.05 was considered statistically significant.

## 3 Results

### 3.1 Study selection

**Figure 1:**
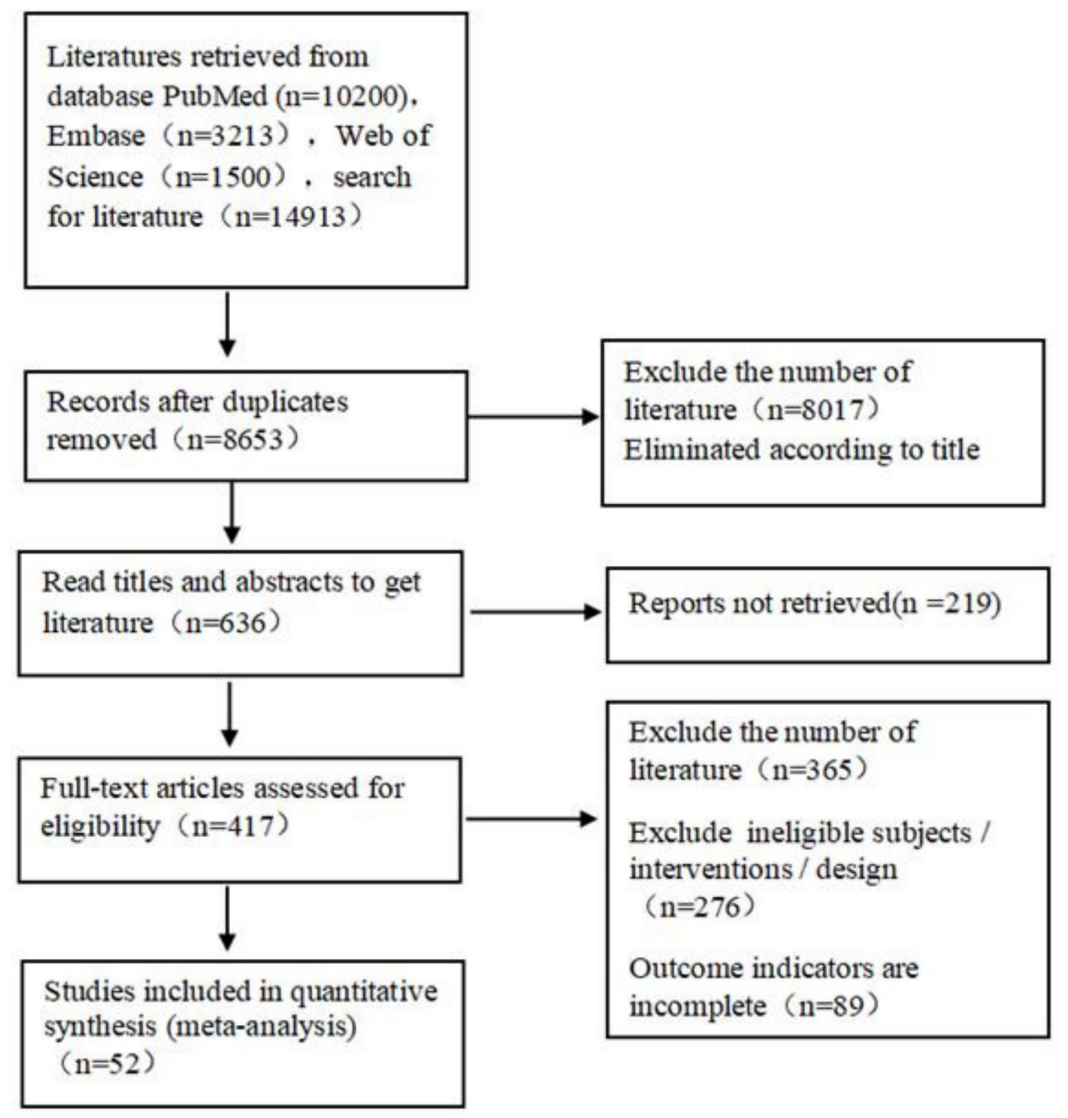
Flow diagram of the study selection process. A total of 14,913 records were identified, 6,260 duplicates were removed, 417 full-text articles were assessed for eligibility, and 52 studies were included in the quantitative synthesis (meta-analysis).

A comprehensive search identified 14,913 records. After removal of 6,260 duplicates, 8,653 records remained for title and abstract screening. A total of 8,017 records were excluded during screening, and 636 reports were sought for retrieval. Of these, 219 reports were not retrieved, leaving 417 full-text articles assessed for eligibility. Among the full-text articles, 365 were excluded, including 276 due to ineligible participants, interventions, or study design and 89 due to incomplete outcome data. Ultimately, 52 studies from 19 countries across six WHO regions were included in the quantitative synthesis (meta-analysis) (Figure 1).

### 3.2 Characteristics of included studies

Among the 52 articles analyzed, 17 studies reported findings from 8 countries in the European Region, involving 6,508,826 individuals and 201,354 cases of Alzheimer's disease (AD). Fifteen studies contributed data from 3 countries in the Western Pacific Region, with 106,211 participants and 2,290 AD cases. In the Region of the Americas, 10 studies across 3 countries included 59,376 participants and 10,365 AD cases. Six studies from 2 countries in the Eastern Mediterranean Region included 17,173 participants and 525 AD cases. Two studies from 1 country in the South-East Asia Region accounted for 29,614 participants and 94 AD cases, while 2 studies from 2 countries in the African Region included 1,520 participants and 44 AD cases. Detailed characteristics of the included studies are presented in Supplementary Table S3.

### 3.3 Risk of bias assessment

Among the 52 included studies, 49 (94.2%) were rated as moderate or high quality, while three (5.8%) were classified as low quality according to the JBI Critical Appraisal Checklist for Prevalence Studies. The full study-level risk-of-bias assessment is available in Supplementary Table S2.

### 3.4 Pooled prevalence and subgroup analyses

#### 3.4.1 Overall pooled prevalence and model sensitivity

**Figure 2:**
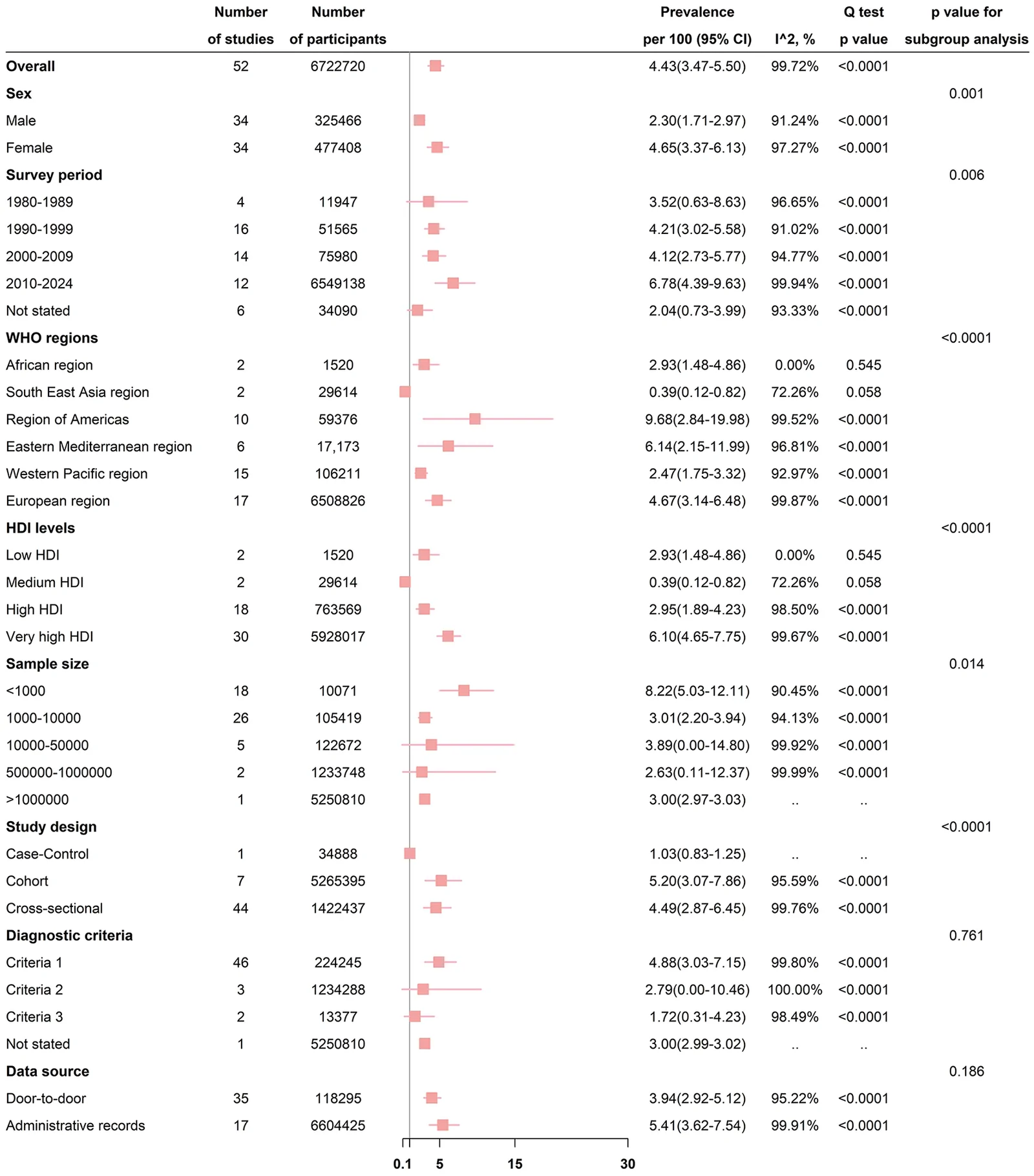
Forest plot of the overall and subgroup pooled reported prevalence of Alzheimer's disease. Results are presented by sex, survey period, World Health Organization region, Human Development Index level, sample size, study design, diagnostic criteria, and data source. Markers represent pooled prevalence estimates, and horizontal lines indicate 95% confidence intervals. I^2^ and Q-test *p*-values describe within-subgroup heterogeneity, whereas *p*-values for subgroup analysis indicate differences between subgroups. Estimates were calculated using a random-effects model with the Freeman–Tukey double-arcsine transformation and were back-transformed to prevalence per 100 population.

Across the 52 included studies, involving 6,722,720 participants, the pooled reported prevalence of Alzheimer's disease was 4.43 per 100 population (95% CI 3.47–5.50, *p* < 0.0001) (Figure 2). As a sensitivity analysis, the pooled reported prevalence was recalculated using a generalized linear mixed model with a binomial likelihood and logit link. The GLMM yielded a pooled reported prevalence of 3.54 per 100 population (95% CI 2.64–4.72), which was lower than the Freeman-Tukey estimate of 4.43 per 100 population (95% CI 3.47–5.50). These results were of the same general order of magnitude, but the lower central estimate from the GLMM indicates that the pooled reported prevalence estimate was somewhat sensitive to the statistical model used (Supplementary Table S5).

Between-study heterogeneity was extremely high (I^2^ = 99.72%), indicating that the pooled estimate represents a statistical summary of highly diverse reported prevalence estimates rather than a precise estimate of a single underlying global prevalence. The included studies varied substantially in survey period, country, WHO region, diagnostic criteria, data source, sample size, study design, and population structure. Consequently, the subsequent subgroup analyses are interpreted primarily as exploratory comparisons of reported prevalence patterns.

#### 3.4.2 Sex-specific and temporal subgroup analyses

In sex-specific analyses, the pooled reported prevalence was higher among females than males. The prevalence estimate was 4.65 per 100 population among females (95% CI 3.37–6.13; 34 studies) and 2.30 per 100 population among males (95% CI 1.71–2.97; 34 studies), with a statistically significant subgroup difference (p = 0.001). However, substantial heterogeneity was observed in both sex-specific analyses, with I^2^ values of 97.27% for females and 91.24% for males. These results indicate that sex-related differences in reported prevalence should be interpreted with caution, as they may also reflect differences in age distribution, survival, study populations, and diagnostic methods (see Supplementary Figures S3A–C).

By survey period, the reported prevalence estimates were 3.52 per 100 population for 1980–1989 (95% CI 0.63–8.63; 4 studies), 4.21 per 100 for 1990–1999 (95% CI 3.02–5.58; 16 studies), 4.12 per 100 for 2000–2009 (95% CI 2.73–5.77; 14 studies), and 6.78 per 100 for 2010–2024 (95% CI 4.39–9.63; 12 studies). Six studies with unstated survey periods yielded a pooled reported prevalence of 2.04 per 100 population (95% CI 0.73–3.99). The subgroup difference by survey period was statistically significant (*p* = 0.006). Although the reported prevalence appeared higher in the most recent period, this pattern should not be interpreted as definitive evidence of a global increase in underlying AD prevalence over time. Studies from different survey periods varied in geographic coverage, diagnostic criteria, data sources, sample sizes, healthcare access, case ascertainment, and population age structure, all of which may have contributed to the observed temporal pattern.

#### 3.4.3 Geographic and HDI subgroup analyses

Reported prevalence estimates varied across WHO regions (*p* < 0.0001). The highest pooled reported prevalence was observed in the Region of the Americas (9.68 per 100 population, 95% CI 2.84–19.98; 10 studies), followed by the Eastern Mediterranean Region (6.14 per 100, 95% CI 2.15–11.99; 6 studies), the European Region (4.67 per 100, 95% CI 3.14–6.48; 17 studies), the African Region (2.93 per 100, 95% CI 1.48–4.86; 2 studies), the Western Pacific Region (2.47 per 100, 95% CI 1.75–3.32; 15 studies), and the South-East Asia Region (0.39 per 100, 95% CI 0.12–0.82; 2 studies). Most regional subgroup estimates showed substantial heterogeneity, particularly in the Region of the Americas (I^2^ = 99.52%), the European Region (I^2^ = 99.87%), the Eastern Mediterranean Region (I^2^ = 96.81%), and the Western Pacific Region (I^2^ = 92.97%). Additionally, several regions were represented by only a few studies. Therefore, regional comparisons should be considered descriptive rather than definitive evidence of differences in disease burden (see Supplementary Figure S2B).

**Figure 3:**
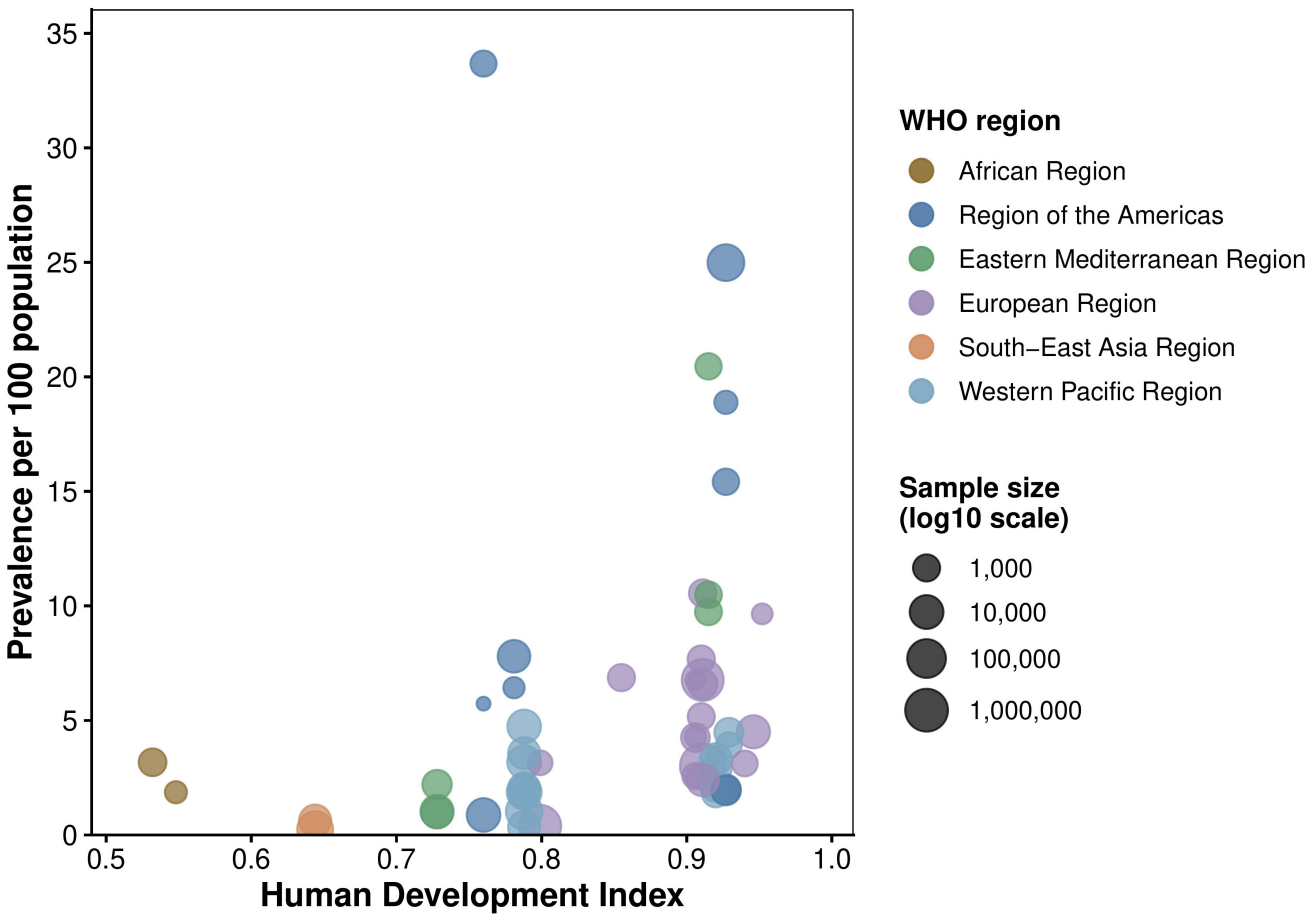
Bubble plot of reported Alzheimer's disease prevalence by Human Development Index. Each bubble represents one study; bubble size represents sample size on a log10 scale, and bubble color indicates the World Health Organization region. The x-axis shows the Human Development Index, and the y-axis shows reported prevalence per 100 population.

Analyses by Human Development Index (HDI) indicated that reported prevalence estimates differed across HDI categories (*p* < 0.0001). The pooled reported prevalence was 6.10 per 100 population in very high-HDI countries (95% CI 4.65–7.75; 30 studies), 2.95 per 100 in high-HDI countries (95% CI 1.89–4.23; 18 studies), 2.93 per 100 in low-HDI countries (95% CI 1.48–4.86; 2 studies), and 0.39 per 100 in medium-HDI countries (95% CI 0.12–0.82; 2 studies). Because the estimates were not strictly monotonic across HDI categories and the low- and medium-HDI groups included few studies, these findings should not be interpreted as evidence of a direct epidemiological gradient in AD risk. Differences in diagnostic capacity, healthcare access, case ascertainment, health-record completeness, life expectancy, population age structure, and data availability may have influenced the observed HDI-related differences (see Figure 3 and Supplementary Figure S2A).

#### 3.4.4 Methodological subgroup analyses

Prevalence estimates also varied by sample size (*p* = 0.014). Studies with fewer than 1,000 participants reported the highest pooled prevalence estimate (8.22 per 100 population, 95% CI 5.03–12.11; 18 studies). Lower estimates were observed among studies with sample sizes of 1,000–10,000 participants (3.01 per 100, 95% CI 2.20–3.94; 26 studies), 10,000–50,000 participants (3.89 per 100, 95% CI 0.00–14.80; 5 studies), 50,000–1,000,000 participants (2.63 per 100, 95% CI 0.11–12.37; 2 studies), and more than 1,000,000 participants (3.00 per 100, 95% CI 2.97–3.03; 1 study). These findings suggest that sample size may have influenced reported prevalence estimates, although the very high heterogeneity in several sample-size categories indicates considerable uncertainty.

When stratified by study design, the pooled reported prevalence was 1.03 per 100 population in case-control studies (95% CI 0.83–1.25; 1 study), 5.20 per 100 in cohort studies (95% CI 3.07–7.86; 7 studies), and 4.49 per 100 in cross-sectional studies (95% CI 2.87–6.45; 44 studies). The subgroup difference by study design was statistically significant (*p* < 0.0001). However, as only one case-control study was included and heterogeneity remained high among cohort and cross-sectional studies, these comparisons should be interpreted with caution.

Subgroup analyses by diagnostic criteria did not show a statistically significant difference in reported prevalence estimates (*p* = 0.761). Studies classified as Criterion 1 reported a pooled prevalence of 4.88 per 100 population (95% CI 3.03–7.15; 46 studies). The corresponding estimates were 2.79 per 100 for Criterion 2 (95% CI 0.00–10.46; 3 studies), 1.72 per 100 for Criterion 3 (95% CI 0.31–4.23; 2 studies), and 3.00 per 100 for studies with not stated diagnostic criteria (95% CI 2.99–3.02; 1 study). Because several diagnostic-criteria categories included few studies and broad diagnostic categories may encompass different clinical frameworks and coding systems, this subgroup analysis should be considered exploratory.

Reported prevalence estimates also differed by data source, although the subgroup difference was not statistically significant (*p* = 0.186). Door-to-door studies reported a pooled prevalence of 3.94 per 100 population (95% CI 2.92–5.12; 35 studies), while studies based on administrative records reported a prevalence of 5.41 per 100 population (95% CI 3.62–7.54; 17 studies). Both categories exhibited substantial heterogeneity, particularly administrative-record studies (I^2^ = 99.91%). Thus, differences by data source may reflect variation in case detection, diagnostic documentation, healthcare utilization, and population coverage rather than differences in underlying disease frequency alone.

### 3.5 Meta-regression analyses

Among the 52 included studies, 46 reported sufficient survey-year information and were included in the survey-year meta-regression analyses. In univariable random-effects meta-regression, survey year was not significantly associated with reported AD prevalence (β per 10-year increase = 0.010, 95% CI: −0.327 to 0.346, *p* = 0.955). After adjustment for WHO region, diagnostic criteria, and log-transformed sample size, survey year remained non-significant (β per 10-year increase = 0.081, 95% CI: −0.273 to 0.434, *p* = 0.654). The multivariable model explained 18.31% of between-study heterogeneity, but substantial residual heterogeneity remained (Supplementary Figure S4).

### 3.6 Sensitivity analysis and publication bias

Sensitivity analyses indicated that exclusion of the three studies classified as low quality did not materially alter the pooled reported prevalence estimate. The leave-one-out sensitivity analysis also demonstrated that no single study alone influenced the overall pooled estimate (Supplementary Figure S5). However, between-study heterogeneity remained extremely high after sequential exclusion of individual studies, suggesting that the observed heterogeneity was not attributable to a single influential study but reflected broader differences across the included evidence base.

Funnel plots indicated mild visual asymmetry in the distribution of effect sizes. Egger's tests did not provide statistically significant evidence of publication bias in either analysis, including analyses that excluded small-sample studies (*p* = 0.9308) and those that included small-sample studies (*p* = 0.6377). However, given the extreme heterogeneity and wide variation in study size, region, diagnostic criteria, and data source, these tests should be interpreted with caution. The absence of statistical significance in Egger's test does not fully rule out small-study effects or selective publication, particularly in subgroup analyses with few studies.

Overall, these sensitivity analyses supported the numerical stability of the pooled estimate. However, the persistently high heterogeneity, mild funnel plot asymmetry, and low certainty of evidence according to GRADE suggest that the pooled prevalence estimate should be interpreted with caution. The certainty of evidence for the primary pooled prevalence estimate was rated as low, primarily due to serious inconsistency and serious indirectness (Supplementary Table S4).

## 4 Discussion

This systematic review and meta-analysis synthesized evidence on the reported prevalence of Alzheimer's disease (AD) across more than four decades and diverse geographic settings. The pooled estimate indicated a substantial reported prevalence of AD among the included studies; however, the extremely high between-study heterogeneity indicates that this estimate should not be interpreted as a precise global prevalence. Rather, it represents a statistical summary of reported prevalence estimates derived from studies that varied considerably in population structure, diagnostic criteria, survey period, data source, sample size, study design, and healthcare context. This finding is consistent with previous global burden estimates indicating that AD and dementia remain substantial public-health challenges in aging populations (13, 14).

Subgroup analyses showed variation in reported prevalence across survey periods, WHO regions, HDI categories, sex, sample size, study design, diagnostic criteria, and data source. These patterns help identify where reported estimates differed across the included evidence base, but they should not be interpreted as direct causal evidence of differences in underlying disease burden. Comparisons across time periods and HDI categories may be affected by changes in diagnostic definitions, case ascertainment, survival, age structure, healthcare access, data availability, and study methodology. Previous reviews of long-term trends in dementia have raised similar concerns, noting that changes in incidence, survival, and diagnostic practices may yield inconsistent prevalence patterns across settings (8).

A major challenge in interpreting temporal patterns is the lack of direct longitudinal comparability across studies. The studies included from different survey periods were not repeated assessments of the same populations; instead, they were conducted in different countries and populations, employing varying diagnostic criteria, case-identification methods, data sources, and sampling strategies. Consequently, the higher reported prevalence in the most recent survey period should not be regarded as definitive evidence of a global increase in underlying AD prevalence over time. This pattern may instead reflect improved diagnostic capacity, expanded access to specialist assessment, changes in diagnostic definitions, more comprehensive health records, longer survival after diagnosis, and differences in population age structure across study periods (15). These factors are particularly relevant in a review spanning more than four decades, during which AD diagnostic frameworks have changed substantially (16, 17).

The findings related to HDI also warrant cautious interpretation. Although reported prevalence estimates were highest in very high-HDI countries, HDI is an ecological, country-level indicator and does not allow for inference of individual-level AD risk. Countries with higher HDI typically have older population structures, longer life expectancy, greater access to specialist care, more comprehensive medical records, and enhanced diagnostic capacity. Previous research has documented differences in AD diagnosis and management across countries (9). In lower-resource settings, diagnosing AD is particularly challenging, as accurate case identification requires clinical expertise, cognitive assessment, and adequate diagnostic infrastructure (18). In contrast, more developed healthcare systems are more likely to detect and record cases across the AD continuum (19). Thus, observed differences across HDI categories may reflect variations in case ascertainment and data availability, as well as potential epidemiological differences. These findings should not be interpreted as evidence that higher socioeconomic development directly increases AD risk.

Contextual factors such as economic development, urbanization, and lifestyle may be relevant when interpreting HDI-related patterns; however, these explanations remain hypothesis-generating within the scope of this review. Previous studies have identified physical inactivity as a potential factor associated with AD risk (20). Tobacco use has also been examined in relation to AD risk (21). Cardiometabolic factors, including diabetes, obesity, hypertension, and cholesterol-related conditions, may contribute to cognitive decline and AD (22). Stress and dietary factors have similarly been discussed as potentially relevant contextual influences (23). These factors were not directly assessed at the individual level in this meta-analysis and should therefore be regarded as possible contextual contributors rather than established explanations for the observed HDI subgroup differences.

Sex-specific analyses indicated a higher reported prevalence of AD among females compared to males. This finding aligns with previous evidence suggesting sex differences in dementia and AD prevalence. Women generally have longer life expectancy and are thus more likely to reach older ages, when AD risk is highest (7). Differences in survival after diagnosis, age composition, healthcare-seeking behavior, and study design may also contribute to the observed sex difference. Although biological mechanisms may be involved, this meta-analysis was not designed to distinguish biological effects from demographic and methodological explanations. Mechanistic studies have explored possible biological pathways underlying sex differences in AD (24). Therefore, the observed sex difference should be interpreted as a pattern in reported prevalence rather than as direct evidence of a single causal mechanism.

Differences in the populations represented by various data sources may have contributed to variation in reported prevalence estimates. Studies using administrative records yielded a higher pooled reported prevalence estimate (5.41 per 100) compared to door-to-door studies (3.94 per 100). This discrepancy may be attributable to the methods used to identify cases in each data source. Healthcare facilities, with greater diagnostic resources, are more likely to identify moderate-to-severe cases that meet clinical criteria for Alzheimer's disease (AD) (25). In contrast, door-to-door studies aim to include the broader population but are limited by the effectiveness of initial screening methods, which often identify individuals with mild cognitive impairment who do not meet the clinical criteria for AD and are therefore excluded from prevalence calculations (26).

Variations in diagnostic criteria may have contributed to the observed heterogeneity. The broad diagnostic categories used in this review encompassed different clinical frameworks, coding systems, and diagnostic eras. For instance, clinical diagnosis may involve various versions of NINCDS-ADRDA, DSM-based criteria, NIA-AA criteria, or other clinical approaches, each with distinct sensitivities and specificities. Major revisions in AD diagnostic frameworks over time may therefore affect the comparability of prevalence estimates across decades and regions (16, 17). This limitation likely contributed to the persistent heterogeneity within diagnostic subgroups.

Age structure represents a significant source of uncertainty. As age is the primary risk factor for AD, variations in age composition across countries, survey periods, Human Development Index (HDI) categories, and data sources can substantially affect prevalence estimates. Many included studies did not provide age-standardized prevalence estimates, and there was considerable variation in age range, age distribution, and reporting detail among the available studies. Consequently, subgroup comparisons across regions, time periods, and HDI categories may partially reflect differences in population age structure rather than true differences in disease frequency. This limitation is especially pertinent when interpreting temporal and HDI-related patterns, given that population aging and age-structure differences can significantly influence estimates of dementia and AD burden (27).

Sensitivity analyses indicated that the pooled estimate remained numerically stable following both leave-one-out analysis and the exclusion of the three low-quality studies. Despite these adjustments, heterogeneity remained extremely high, suggesting that the observed variability likely reflects substantial differences across the evidence base rather than the impact of a single outlying study. Funnel plots demonstrated mild visual asymmetry, although Egger's tests did not reach statistical significance. Given the extreme heterogeneity and substantial variation in study size, region, diagnostic approach, and data source, the lack of statistical significance in Egger's test does not definitively exclude the possibility of small-study effects or selective publication (28). These results underscore the need for caution when interpreting the pooled estimates.

This review is subject to several limitations. First, substantial heterogeneity among the included studies limits the interpretability of a single pooled prevalence estimate. Second, variations in diagnostic criteria, case-identification procedures, data sources, and healthcare access across countries and survey periods may have introduced misclassification and reduced comparability. Third, some subgroup analyses were based on a limited number of studies, particularly within certain WHO regions and HDI categories, which reduces the stability of these subgroup estimates. Fourth, the absence of age-standardized prevalence estimates in some studies and differences in age structure may have influenced comparisons across regions, periods, and HDI categories. Fifth, the inclusion of only English-language publications may have introduced language bias and resulted in the omission of relevant studies published in other languages. Sixth, the lack of a dedicated gray literature search means that unpublished estimates, government reports, or local surveillance data may not have been fully captured. Finally, as most included studies were cross-sectional or observational, this review cannot establish causal relationships underlying the observed temporal, regional, HDI-related, or sex-specific differences.

Despite these limitations, this review offers a comprehensive synthesis of reported AD prevalence across multiple decades and diverse geographic settings. The findings underscore the need for more standardized and comparable population-based epidemiological studies, particularly in underrepresented and lower-resource settings. Future research should employ clearer and more consistent diagnostic criteria, report age-standardized estimates when feasible, provide transparent information on data sources and case ascertainment, and enhance representation from regions with limited current evidence. These efforts are essential for improving the comparability of global AD prevalence estimates and for informing surveillance, prevention, and health policy planning.

## 5 Conclusions

This systematic review and meta-analysis synthesized evidence on the reported prevalence of Alzheimer's disease from 1980 to 2024 and found substantial variation across survey periods, WHO regions, HDI levels, sex, and methodological characteristics. Reported prevalence estimates differed across the survey periods examined, but meta-regression did not identify a significant association with survey year. Therefore, the available evidence does not support a consistent global increase in Alzheimer's disease prevalence over time. Given the extremely high between-study heterogeneity, the pooled estimates should be interpreted as summaries of reported prevalence across diverse study contexts rather than as precise estimates of a single global disease frequency. Future studies should use more standardized diagnostic criteria, age-comparable prevalence reporting, transparent case-ascertainment procedures, and broader geographic coverage, particularly in underrepresented and lower-resource settings, to improve the comparability of AD prevalence estimates and support global surveillance and health policy planning.

## Data Availability

The combined dataset used for the meta-analysis and the R code are not publicly deposited. The data were extracted from previously published studies and are available from Mengqi Hu upon reasonable request. Access is restricted to academic and non-commercial use, with appropriate citation of the original studies and this article. Requests to access the dataset and R code should be directed to Mengqi Hu at 15665832356@163.com.
