# Supplementary material for: Temporal trends in the prevalence of Alzheimer's disease, 1980–2024: a systematic review and meta-analysis
Source: Front Public Health. 2026 Aug 19;14:1884058. doi: 10.3389/fpubh.2026.1884058 (PMC13533708; doi:10.3389/fpubh.2026.1884058)
Supplement: Supplementary file 2 [file Data_Sheet_2.pdf]

Supplementary Table S2. Evaluation of bias risk utilizing the Joanna Briggs Institute (JBI) Critical Appraisal Checklist for Research on Prevalence Data

Study	Q1	Q2	Q3	Q4	Q5	Q6	Q7	Q8	Q9	JBI quality
Silke Kern ry	yes	yes	unclear	yes	yes	yes	yes	yes	yes	High
Ana Lopez |2023	yes	no	yes	yes	yes	yes	unclear	yes	unclear	Moderate
KEIKO HATADA |1999	yes	yes	yes	yes	yes	yes	yes	yes	yes	High
Jianping Jia |2014	yes	yes	yes	yes	yes	yes	yes	yes	yes	High
Ivan Dimitrov |2012	yes	yes	yes	yes	yes	yes	unclear	yes	yes	High
W.A.Rocca |1990	yes	yes	yes	yes	yes	yes	yes	yes	yes	High
Evgeniy |2024	yes	no	yes	yes	yes	yes	unclear	yes	unclear	Moderate
Alewin | 1995	yes	yes	yes	yes	yes	yes	yes	yes	unclear	High
A.Bowirrat |2001	yes	yes	yes	yes	yes	yes	yes	yes	yes	High
Zhou DF |2006	yes	yes	yes	yes	yes	yes	yes	yes	yes	High
Y.Obadia |1997	yes	yes	yes	yes	yes	yes	yes	yes	yes	High
Tarek |2014	yes	yes	yes	yes	yes	yes	unclear	no	yes	Moderate
Hamdy |2012	yes	yes	yes	yes	yes	yes	yes	no	yes	High
Ks Hall |2009	yes	no	yes	yes	yes	yes	yes	yes	unclear	Moderate
J in Hyeong Jhoo |2008	yes	yes	yes	yes	no	yes	yes	yes	unclear	Moderate
Jong Inn Woo |1998	yes	yes	yes	yes	no	yes	yes	yes	yes	High
TATSUO YAMADA |2002	yes	yes	no	yes	no	yes	unclear	no	unclear	Low
Astrid |2024	yes	yes	yes	yes	yes	yes	yes	yes	yes	High
Ricardo |2020	no	no	yes	yes	yes	yes	unclear	yes	unclear	Moderate
M D |2002	yes	yes	no	yes	yes	yes	yes	yes	yes	High
Anne |2012	yes	yes	yes	yes	yes	yes	yes	yes	unclear	High
Stella-Maria |2014	yes	yes	yes	yes	yes	yes	yes	yes	yes	High
H C |1995	yes	yes	yes	yes	yes	yes	unclear	yes	yes	High
T Yamada |2001	yes	yes	yes	yes	no	yes	yes	no	unclear	Low
Mitsuru |1999	yes	yes	no	yes	yes	yes	unclear	no	yes	Low
H. Gurvit |2008	yes	yes	yes	yes	yes	yes	yes	yes	yes	High
V. C |1998	yes	yes	yes	yes	yes	yes	yes	yes	yes	High
Cristorfer |2024	yes	yes	yes	yes	yes	yes	unclear	yes	unclear	Moderate
Abdulkareem |2011	yes	yes	yes	yes	yes	yes	yes	yes	yes	High
Cosme |2004	yes	no	yes	yes	yes	yes	yes	yes	yes	High
Desheng wang |2011	yes	yes	yes	yes	yes	yes	yes	yes	yes	High
Miguel |2013	yes	yes	yes	yes	yes	yes	yes	yes	yes	High
M. Ikeda |2001	yes	yes	yes	yes	yes	yes	yes	no	yes	High
Natan |2021	yes	yes	yes	yes	yes	no	unclear	yes	unclear	Moderate
Tognoni |2005	yes	yes	yes	yes	yes	yes	yes	yes	yes	High
Sekita A|2010	yes	yes	yes	yes	yes	yes	yes	yes	yes	High
LENNON |2022	yes	no	yes	yes	yes	yes	yes	yes	unclear	Moderate
Fang | 2013	yes	no	yes	yes	yes	yes	unclear	yes	unclear	Moderate
Kathleen |2000	yes	yes	yes	yes	yes	yes	yes	yes	yes	High
wenzhzi |2000	yes	yes	yes	yes	yes	yes	yes	yes	yes	High
Ch |2001	yes	no	yes	yes	yes	yes	yes	yes	yes	High

R.I |1987	yes	yes	yes	yes	yes	yes	yes	yes	yes	High
Mingyuan |1990	yes	yes	yes	yes	yes	yes	yes	yes	yes	High
JM | 1995	yes	yes	yes	yes	yes	yes	yes	yes	yes	High
Teresa |2022	yes	yes	yes	yes	yes	yes	yes	yes	yes	High
GILL |1990	yes	yes	yes	yes	yes	yes	yes	yes	yes	High
A. Sicras |2005	yes	yes	yes	yes	yes	yes	yes	yes	yes	High
Abdul |1998	yes	yes	yes	yes	yes	yes	yes	yes	yes	High
M P |1996	yes	yes	yes	yes	yes	yes	yes	yes	yes	High
R | 2009	yes	yes	yes	yes	yes	yes	unclear	yes	yes	High
R | 2015	yes	yes	yes	yes	yes	yes	yes	yes	yes	High
Cheng | 2022	yes	yes	yes	yes	yes	yes	yes	yes	yes	High
