# Supplementary material for: Temporal trends in the prevalence of Alzheimer's disease, 1980–2024: a systematic review and meta-analysis
Source: Front Public Health. 2026 Aug 19;14:1884058. doi: 10.3389/fpubh.2026.1884058 (PMC13533708; doi:10.3389/fpubh.2026.1884058)
Supplement: Supplementary file 3 [file Data_Sheet_3.pdf]

Supplementary Table S3. Study characteristics, prevalence estimates, and quality assessment of included studies

Author(year)	Country;WHO region;HDI	Survey period	Data source	Study design	Diagnostic criteria	Age range;years	Females(%)	Sample size	Reported prevalence of AD per 100 population	JBI quality assessment
Silke Kern |2018	Sweden;European region;very high	2014-2016	Administrative records	Cross-sectional	1	≥70	49.8%	259	9.7	High
Ana Lopez |2023	Sweden;European region;very high	Jan,2011-Dec,2020	Administrative records	Cohort	Not stated	≥60	NA	5250810	3	Moderate
KEIKO HATADA |1999	Japan;Western Pacific Region;very high	1995	Administrative records	Cross-sectional	3	≥60	60.4%	3965	6.2	High
Jianping Jia |2014	China;Western Pacific Region;high	Oct,2008-Oct, 2009	Administrative records	Cross-sectional	1	≥65	57.38%	10276	3.21(2.87-3.5 5)	High
Ivan Dimitrov |2012	Bulgaria;European region;high	No stated	Administrative records	Cross-sectional	2	≥65	58.70%	540	3.1(2.3-3.9)	High
W.A.Rocca |1990	Italy;European region;very high	1987	Door-to-door	Cross-sectional	1	≥59	55.90%	778	2.6	High
Evgeniy |2024	Bulgaria;European region;high	2018	Administrative records	Cross-sectional	2	≥30	61.67%	642013	0.39	Moderate
Alewin | 1995	Netherlands;European region;very high	1990	Administrative records	Cross-sectional	1	≥55	77%	7528	4.5	High
A.Bowirrat |2001	Israel;Eastern Mediterranean Region;very high	1995	Door-to-door	Cross-sectional	1	≥60	55.90%	821	20.5	High
Zhou DF |2006	China;Western Pacific Region;high	Nov, 1999-Apr ,2000	Door-to-door	Cross-sectional	1	≥50	59.60%	16095	1.83	High
Y.Obadia |1997	France;European region;very high	1991	Door-to-door	Cross-sectional	1	≥70	39.55%	1608	5.5(4.0–6.9)	High
Tarek |2014	Egypt;Eastern Mediterranean Region;high	Jul,2009-Jan,2012	Door-to-door	Cross-sectional	1	≥50	44%	4329	0.97	Moderate
Hamdy |2012	Egypt;Eastern Mediterranean Region;high	Apr,2005-Jul,2008	Door-to-door	Cross-sectional	1	≥50	45.50%	8173	1.03	High
Ks Hall |2009	USA;Region of the Americas;very high	1992-2001	Door-to-door	Cross-sectional	1	≥65	65.33%	2212	5.47	Moderate
J in Hyeong Jhoo |2008	South Korea;Western Pacific Region;very high	Sep,2005-Aug, 2006	Administrative records	Cohort	1	≥65	57.84%	714	4.8(3.3–6.4)	Moderate
Jong Inn Woo |1998	South Korea;Western Pacific Region;very high	Aug, 1992-Sep, 1993	Door-to-door	Cross-sectional	1	≥65	53.82%	1674	4.5	High
TATSUO YAMADA |2002	Brazil;Region of the Americas;high	2000	Door-to-door	Cross-sectional	1	≥70	55.41%	157	5.7	Low
Astrid |2024	USA;Region of the Americas;very high	2010	Door-to-door	Cohort	1	≥72	70.02%	397	18.9	High
Ricardo |2020	Brazil;Region of the Americas;high	2008-2015	Administrative records	Cross-sectional	1	≥18	61.44%	760	33.7	Moderate
M D |2002	Italy;European region;very high	1996	Administrative records	Cross-sectional	1	≥74	63.96%	222	6.7	High
Anne |2012	Israel;Eastern Mediterranean Region;very high	Jan,2003-Dec, 2008	Door-to-door	Cohort	1	≥65	50.60%	944	9.8(8.0- 10.2)	High
Stella-Maria |2014	Tanzania;African Region;low	Apr-Sep,2010	Door-to-door	Cross-sectional	1	≥70	56. 17%	1198	3.7(2.5-4.9)	High
H C |1995	China(Taiwan);Western Pacific Region;high	1988-1989	Door-to-door	Cross-sectional	1	≥41	48.02%	5297	0.34	High
T Yamada Mitsuru |2001	Japan;Western Pacific Region;very high	1998	Door-to-door	Cross-sectional	1	≥65	59.54%	3715	2.1	Low
Mitsuru |1999	Japan;Western Pacific Region;very high	1995	Door-to-door	Cross-sectional	1	≥65	54.22%	201	3.5	Low
H. Gurvit |2008	Turkey;European region;very high	No stated	Door-to-door	Cross-sectional	1	≥70	61.20%	1019	16.0(11.0-21.0)	High
V. C |1998	India;Southeast Asian region;medium	No stated	Door-to-door	Cross-sectional	1	≥55	46.91%	5126	0.62(0.43-0.88)	High
Cristorfer |2024	Spain;European region;very high	2000-2021	Administrative records	Cross-sectional	2	≥60	Not stated	591735	6.77	Moderate
Abdulkareem |2011	Nigeria;African Region;low	2007	Door-to-door	Cross-sectional	1	≥65	60.24%	322	1.86(0.37-3.35)	High
Cosme|2004	Mexico;Region of the Americas;high	2000-2002	Door-to-door	Cross-sectional	1	≥50	76.07%	280	6.4	High
Desheng wang |2011	China;Western Pacific Region;high	Jun,2008-Jun, 2009	Door-to-door	Cross-sectional	1	≥55	56%	9266	4.79	High
Miguel |2013	Spain;European region;very high	Feb,2009-May ,2010	Administrative records	Cross-sectional	1	≥65	57%	2170	6.6	High

M.Ikeda |2001	Japan;Western Pacific Region;very high	Jan, 1997-Mar, 1998	Door-to-door	Cross-sectional	1	≥65	Not stated	1162	1.8	High
Natan|2021	Brazil;Region of the Americas;high	2015-2016	Administrative records	Cohort	3	≥50	Not stated	9412	0.75(0.58-0.98)	Moderate
Tognoni |2005	Italy;European region;very high	Apr,2000-Dec,2000	Door-to-door	Cross-sectional	1	≥65	59.62%	1600	4.2	High
Sekita.A|2010	Japan;Western Pacific Region;very high	1985-2000	Door-to-door	Cross-sectional	1	≥65	Not stated	5076	1.1	High
LENNON |2022	USA;Region of the Americas;very high	Sep,2005-Mar, 2020	Administrative records	Cross-sectional	1	No stated	50.63%	36925	24.9	Moderate
Fang |2013	China;Western Pacific Region;high	2002-2012	Administrative records	Case-Control	1	≥60	44.42%	34888	1.03	Moderate
Kathleen |2000	USA;Region of the Americas;very high	No stated	Door-to-door	Cohort	1	≥65	Not stated	2212	1.94	High
Wenzhzi |2000	China;Western Pacific Region;high	1995	Door-to-door	Cross-sectional	1	≥65	56.54%	3728	1.85(1.44-2.35)	High
Ch |2001	India;Southeast Asian region;medium	No stated	Door-to-door	Cross-sectional	1	≥40	51.50%	24488	0.25	High
R.I |1987	USA;Region of the Americas;very high	Jul, 1980-Jun, 1982	Door-to-door	Cross-sectional	1	≥65	48.34%	817	15.3(13.0–17.5)	High
Ming yuan|1990	China;Western Pacific Region;high	1987	Door-to-door	Cross-sectional	1	≥55	56.28%	5055	2.99	High
JM |1995	Spain;European region;very high	1991	Door-to-door	Cross-sectional	1	≥70	50. 13%	1127	10.56	High
Teresa|2022	Mexico;Region of the Americas;high	2017-2018	Administrative records	Cross-sectional	1	≥60	Not stated	6204	7.8	High
GILL|1990	UK;European region;very high	No stated	Door-to-door	Cross-sectional	1	≥65	Not stated	705	3.1	High
A. Sicras |2005	Spain;European region;very high	2003	Administrative records	Cross-sectional	1	≥64	58%	6004	2.4	High
Abdul |1998	Egypt; Eastern Mediterranean Region; high	1993- 1994	Door-to-door	Cross-sectional	1	≥60	48.65%	2000	2.2	High
M P|1996	Italy; European region;very high	1995	Door-to-door	Cross-sectional	1	≥64	56.81%	968	5.2(3.8-6.6)	High
R | 2009	Italy; European region; very high	2005-2006	Door-to-door	Cross-sectional	1	≥60	57. 14%	280	4.1	High
R | 2015	Israel;Mediterranean Region; very high	2003-2012	Door-to-door	Cohort	1	≥65	50.22%	906	10	High
Cheng |2022	China; Western Pacific Region; high	2018	Door-to-door	Cross-sectional	1	≥60	57.10%	5096	3.57(3.08–4.12)	High

Note. Prevalence estimates are presented as reported prevalence of AD per 100 population. When 95% CIs were reported in the original articles, they are shown in parentheses; otherwise, only point estimates are presented. For studies reporting multiple diagnostic approaches, each study was assigned to one analytical category to avoid double-counting in subgroup analyses. Wenzhzi |2000 reported multiple diagnostic approaches, including clinical diagnostic criteria, and was therefore classified as Criterion 1.
