# Supplementary material for: Temporal trends in the prevalence of Alzheimer's disease, 1980–2024: a systematic review and meta-analysis
Source: Front Public Health. 2026 Aug 19;14:1884058. doi: 10.3389/fpubh.2026.1884058 (PMC13533708; doi:10.3389/fpubh.2026.1884058)
Supplement: Supplementary file 4 [file Data_Sheet_4.pdf]

Supplementary Table S4. GRADE assessment of the certainty of evidence

Quality Assessment								
**No. of Studies**	**No. of Participants**	**Risk of Bias**	**Inconsistency**	**Indirectness**	**Imprecision**	**Publication Bias**	**Prevalence (95% CI)**	**Quality**
52	6,722,720	Not serious	Serious	Serious	Not serious	Not serious	4.43 (3.47–5.50)	Low

CI = confidence interval; JBI = Joanna Briggs Institute.

a. Three studies were classified as low quality according to the JBI assessment. Sensitivity analysis excluding these studies did not materially change the pooled prevalence estimate; therefore, risk of bias was rated as not serious.

b. Inconsistency was rated as serious because between-study heterogeneity was extremely high (I^2 = 99.72%) and remained substantial in sensitivity and subgroup analyses.

c. Indirectness was rated as serious because the included studies covered only 19 countries, with limited representation from low- and medium-HDI settings and substantial variation in age structure, diagnostic criteria, data sources, and case ascertainment.

d. Imprecision was rated as not serious for the primary pooled estimate because the overall 95% CI was relatively narrow. However, several subgroup estimates were based on few studies and had wide confidence intervals; therefore, subgroup findings were interpreted cautiously.

e. Publication bias was rated as not serious after considering Egger's tests, funnel-plot inspection, and the interpretive limitations caused by extreme heterogeneity. Egger's tests were not statistically significant; however, mild funnel-plot asymmetry and potential small-study effects could not be excluded because of extreme heterogeneity and wide variation in study size.
