# Supplementary material for: Temporal trends in the prevalence of Alzheimer's disease, 1980–2024: a systematic review and meta-analysis
Source: Front Public Health. 2026 Aug 19;14:1884058. doi: 10.3389/fpubh.2026.1884058 (PMC13533708; doi:10.3389/fpubh.2026.1884058)
Supplement: Supplementary file 5 [file Data_Sheet_5.pdf]

Supplementary Table S5. Comparison of Freeman-Tukey and GLMM pooled prevalence estimates

Method	Pooled prevalence per 100 **population**	**95% CI lower**	**95% CI upper**
Freeman-Tukey random-effects model	4.43	3.47	5.5
GLMM logistic-normal random-effects model	3.54	2.64	4.72
