# Supplementary material for: Temporal trends in the prevalence of Alzheimer's disease, 1980–2024: a systematic review and meta-analysis
Source: Front Public Health. 2026 Aug 19;14:1884058. doi: 10.3389/fpubh.2026.1884058 (PMC13533708; doi:10.3389/fpubh.2026.1884058)
Supplement: Supplementary file 6 [file Data_Sheet_6.pdf]

Supplementary Text S1. Search Strategy

The literature search was conducted in PubMed, Embase, and Web of Science from database inception to December 31, 2024. The search strategy combined controlled vocabulary terms and free-text terms related to Alzheimer's disease, prevalence, epidemiology, and population-based observational studies. The search was restricted to English-language publications. The detailed database-specific search strategies are provided below.

PubMed

(("Alzheimer Disease"[Mesh] OR "Alzheimer disease"[Title/Abstract] OR "Alzheimer's disease"[Title/Abstract] OR "Alzheimer dementia"[Title/Abstract] OR Alzheimer*[Title/Abstract])AND("Prevalence"[Mesh] OR "Epidemiology"[Subheading] OR prevalence[Title/Abstract] OR epidemiolog*[Title/Abstract] OR frequency[Title/Abstract] OR occurrence[Title/Abstract] OR "population-based"[Title/Abstract] OR "community-based"[Title/Abstract] OR survey*[Title/Abstract] OR "cross-sectional"[Title/Abstract] OR cohort[Title/Abstract] OR registry[Title/Abstract] OR database*[Title/Abstract]))AND English[Language]AND ("1900/01/01"[Date - Publication] : "2024/12/31"[Date - Publication])NOT (animals[MeSH Terms] NOT humans[MeSH Terms])

Embase

('alzheimer disease'/exp OR 'alzheimer disease':ti,ab,kw OR "alzheimer's disease":ti,ab,kw OR 'alzheimer dementia':ti,ab,kw OR alzheimer*:ti,ab,kw) AND('prevalence'/exp OR 'epidemiology'/exp OR prevalence:ti,ab,kw OR epidemiolog*:ti,ab,kw OR frequency:ti,ab,kw OR occurrence:ti,ab,kw OR 'population-based':ti,ab,kw OR 'community-based':ti,ab,kw OR survey*:ti,ab,kw OR 'cross-sectional':ti,ab,kw OR cohort:ti,ab,kw OR registry:ti,ab,kw OR database*:ti,ab,kw)AND [english]/limAND [humans]/limAND [1900-2024]/py

Web of Science

TS = ((Alzheimer* OR "Alzheimer disease" OR "Alzheimer's disease" OR "Alzheimer dementia")AND(prevalence OR epidemiolog* OR frequency OR

occurrence OR "population-based" OR "community-based" OR survey* OR "cross-sectional" OR cohort OR registry OR database*))

Timespan: database inception to December 31, 2024

Language: English

Document type: Article
