# Supplementary material for: Temporal trends in the prevalence of Alzheimer's disease, 1980–2024: a systematic review and meta-analysis
Source: Front Public Health. 2026 Aug 19;14:1884058. doi: 10.3389/fpubh.2026.1884058 (PMC13533708; doi:10.3389/fpubh.2026.1884058)
Supplement: Supplementary file 7 [file Data_Sheet_7.pdf]

Supplementary Text S2. Human Development Index (HDI)

The Human Development Index (HDI) is a composite metric created by the United Nations Development Programme (UNDP) to evaluate the level of human development in different countries. It assesses a country's performance across three key dimensions: longevity, measured by life expectancy at birth; education, measured by mean years of schooling and expected years of schooling; and standard of living, measured by gross national income (GNI) per capita adjusted for purchasing power parity (PPP) in US dollars.

The HDI establishes minimum and maximum thresholds for each dimension, referred to as "goalposts," and indicates a country's position relative to these benchmarks, resulting in a score ranging from 0 to 1. A higher HDI score reflects a higher level of human development.

Countries and territories are categorized into four groups according to the HDI values established by the UNDP: very high HDI (≥0.80), high HDI (0.70–0.79), medium HDI (0.55–0.69), and low HDI (<0.55).
