# Supplementary material for: Temporal trends in the prevalence of Alzheimer's disease, 1980–2024: a systematic review and meta-analysis
Source: Front Public Health. 2026 Aug 19;14:1884058. doi: 10.3389/fpubh.2026.1884058 (PMC13533708; doi:10.3389/fpubh.2026.1884058)
Supplement: Supplementary file 8 [file Data_Sheet_8.pdf]

Supplementary Text S3. Diagnostic criteria for Alzheimer's disease

Studies utilizing different diagnostic standards for Alzheimer's disease (AD) were categorized into four distinct categories: Criterion 1 included clinical diagnostic criteria, such as the NINCDS–ADRDA criteria, which were developed by the National Institute of Neurological Disorders and Stroke and the Alzheimer's Disease and Related Disorders Association, as well as the DSM-5 criteria for diagnosing "neurocognitive disorder due to Alzheimer's disease" and the clinical diagnostic guidelines proposed by the National Institute on Aging and the Alzheimer's Association; Criterion 2 pertained to diagnoses based on International Classification of Diseases codes; Criterion 3 included diagnoses based on the identification of characteristic clinical symptoms; and not-stated criteria included studies in which the diagnostic criteria were not specifically reported.
